# Genome-Wide Inference of Essential Genes in *Dirofilaria immitis* Using Machine Learning

**DOI:** 10.3390/ijms26209923

**Published:** 2025-10-12

**Authors:** Túlio L. Campos, Pasi K. Korhonen, Neil D. Young, Sunita B. Sumanam, Whitney Bullard, John M. Harrington, Jiangning Song, Bill C. H. Chang, Richard J. Marhöfer, Paul M. Selzer, Robin B. Gasser

**Affiliations:** 1 Department of Biosciences, Melbourne Veterinary School, Faculty of Science, The University of Melbourne, Parkville, VIC 3010, Australia; tulio.campos@unimelb.edu.au (T.L.C.); pasi.korhonen@unimelb.edu.au (P.K.K.); nyoung@unimelb.edu.au (N.D.Y.); sunita.sumanam@unimelb.edu.au (S.B.S.); bchang@ozomics.com (B.C.H.C.); 2 Bioinformatics Core Facility, Aggeu Magalhães Institute (Fiocruz), Recife 50740-465, PE, Brazil; 3 Boehringer Ingelheim Animal Health USA Inc., Athens, GA 30601, USA; whitney.bullard.phd@gmail.com (W.B.); john.harrington@boehringer-ingelheim.com (J.M.H.); 4 Faculty of IT, Department of Data Science and AI, Monash University, Clayton, VIC 3800, Australia; jiangning.song@monash.edu; 5 Biomedicine Discovery Institute and Department of Biochemistry and Molecular Biology, Monash University, Clayton, VIC 3800, Australia; 6 Monash Data Futures Institute, Monash University, Clayton, VIC 3800, Australia; 7 Boehringer Ingelheim Animal Health, Binger Strasse 173, 55216 Ingelheim am Rhein, Germany; richard.marhoefer@boehringer-ingelheim.com (R.J.M.); paul.selzer@boehringer-ingelheim.com (P.M.S.)

**Keywords:** machine learning, essential genes, *Dirofilaria immitis*, dirofilariasis, heartworm disease

## Abstract

The filarioid nematode *Dirofilaria immitis* is the causative agent of heartworm disease, a major parasitic infection of canids, felids and occasionally humans. Current prevention relies on macrocyclic lactone-based chemoprophylaxis, but the emergence of drug resistance highlights the need for new intervention strategies. Here, we applied a machine learning (ML)-based framework to predict and prioritise essential genes in *D. immitis* in silico, using genomic, transcriptomic and functional datasets from the model organisms *Caenorhabditis elegans* and *Drosophila melanogaster*. With a curated set of 26 predictive features, we trained and evaluated multiple ML models and, using a defined threshold, we predicted 406 ‘high-priority’ essential genes. These genes showed strong transcriptional activity across developmental stages and were inferred to be enriched in pathways related to ribosome biogenesis, translation, RNA processing and signalling, underscoring their potential as anthelmintic targets. Transcriptomic analyses suggested that these genes are associated with key reproductive and neural tissues, while chromosomal mapping revealed a relatively even genomic distribution, in contrast to patterns observed in *C. elegans* and *Dr. melanogaster*. In addition, initial evidence suggested structural variation in the X chromosome compared with a recently published *D. immitis* assembly, indicating the importance of integrating long-read sequencing with high-throughput chromosome conformation capture (Hi-C) mapping. Overall, this study reinforces the potential of ML-guided approaches for essential gene discovery in parasitic nematodes and provides a foundation for downstream validation and therapeutic target development.

## 1. Introduction

*Dirofilaria immitis* (Leidy, 1856)—a filarioid nematode—is one of the most important parasites of canids and felids, causing heartworm disease (dirofilariasis or dirofilarosis) [1]. From a health perspective, dogs, and to a lesser extent, cats are affected most by this disease, but humans can occasionally become infected when the prevalence of *D. immitis* infection in dogs and cats is high. Some failures of preventative anthelminthic treatments against *D. immitis* infection in dogs, including macrocyclic lactone-based prophylactic drugs, have been reported in the USA, particularly in regions of high heartworm challenge [1,2,3]. This challenge emphasises the need to search for new preventative drugs or a vaccine, built on a solid understanding of the molecular biology in *D. immitis* and/or its relationship with its host animals [4]. Treatment of heartworm disease in the definitive hosts can affect the heartworm developmental cycle from the infective L3 stage to the adult worm (female and male) and microfilariae (L1s), and preventative treatment relies on killing L3s as they enter the definitive host (https://www.heartwormsociety.org/veterinary-resources/american-heartworm-society-guidelines; accessed on 20 August 2025).

Deep exploration of the transcriptomes, genomes and proteomes of *D. immitis* offers an opportunity to unravel the molecular pathways and mechanisms that underpin key biological processes, and, in turn, may provide strategies for the discovery and design of new interventions [4]. Accurate analysis of nucleic acid and protein sequences, typically via comparison with reference organisms, is vital for generating molecular biological insights, with advanced bioinformatic workflows facilitating these analyses and the discovery of new intervention targets.

Traditional approaches to anthelmintic target discovery can often be protracted, expensive and technically demanding [5,6]. To address these limitations, we have investigated the application of in silico strategies for predicting and ranking essential genes that might subsequently be tested as therapeutic targets. Our earlier work [7,8] centred on developing and applying machine learning (ML) frameworks to predict gene essentiality in two of the best-characterised multicellular model organisms: *Caenorhabditis elegans* (a free-living nematode) and *Drosophila melanogaster* (the vinegar fly). These species were chosen because they can be propagated in the laboratory and, importantly, because their chromosome-scale genomes and extensive functional, molecular, physiological and developmental data are openly accessible through curated repositories such as WormBase and FlyBase [9,10,11,12]. The availability of these resources has enabled in-depth analyses of essential genes in both species. In our artificial intelligence (AI)-driven studies [13,14,15], we showed that informative features derived from these data sets could be engineered to allow statistically robust predictions of essential genes within and across *C. elegans* and *Dr. melanogaster*.

In contrast, the inability to maintain the full life cycle of filarioid nematodes in vitro, together with environmental differences between the host/vector and artificial culture systems, has hampered the development of functional genomic assays across parasite life stages and sexes. This limitation has been a major barrier to determining essential genes and prioritising potential drug targets. Having now shown that ML-based bioinformatic pipelines can reliably predict essential genes within and between *C. elegans* and *Dr. melanogaster* [13,14], we anticipate that these approaches can also support cross-species predictions among related nematodes or arthropods (ecdysozoans), provided that appropriate molecular data sets are available. Extending from recent studies on *Haemonchus contortus*, *Brugia malayi* and *Onchocerca volvulus* [16,17], we apply this cross-species ML framework to systematically predict and prioritise essential genes in *D. immitis*, using *C. elegans* and *Dr. melanogaster* as training references, and assess the relationship between gene essentiality and transcriptional activity in *D. immitis*.

## 2. Results

### 2.1. Genome Assembly, Quality of the Assembly, Gene Prediction and Annotation

**Figure 1:**
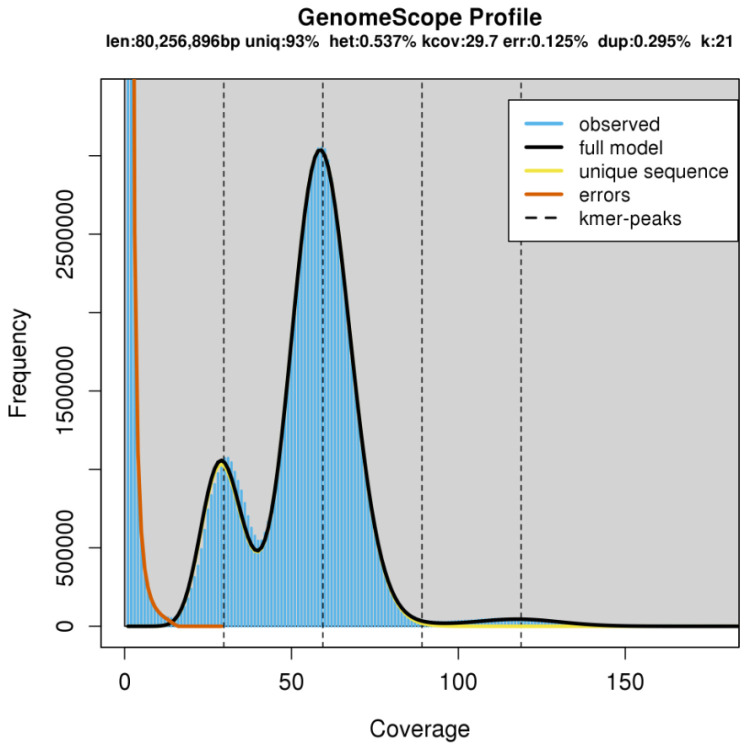
Size of the genome of *Dirofilaria immitis*. To estimate the genome size and assess sequence properties of *D. immitis*, we employed GenomeScope using PacBio high-fidelity (HiFi) sequencing reads. The k-mer profile (k = 21) revealed a genome size of ~80.3 Mb, with an estimated unique sequence content of 93%. The analysis also indicated a low heterozygosity rate of 0.537%, a k-mer coverage depth of ~29.7×, a sequencing error rate of 0.125% and a duplication level of 0.295%. The resultant model, fitted to the observed k-mer frequency histogram, showed clear peaks corresponding to distinct copy number classes, consistent with a predominantly diploid genome. Together, these results support the high quality of the *D. immitis* genome assembly obtained in this study.

**Figure 2:**
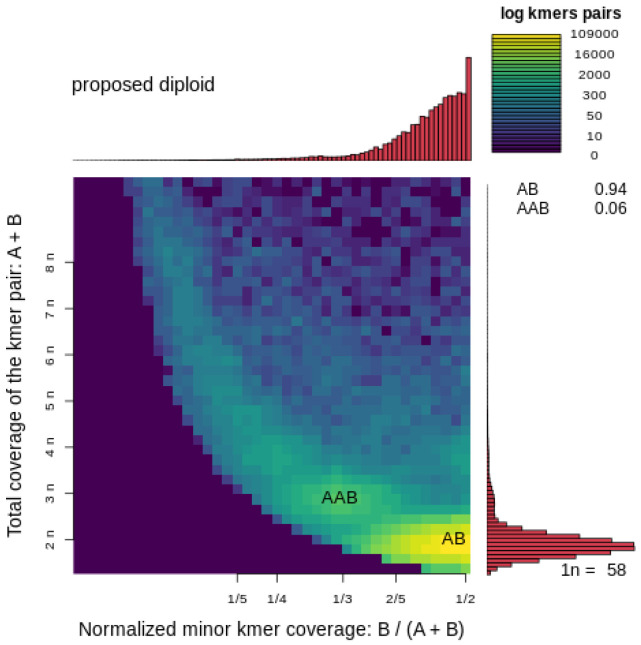
Inference of diploidy in *Dirofilaria immitis* using a SmudgePlot analysis of PacBio high-fidelity (HiFi) sequence data. This SmudgePlot visualises k-mer pair frequencies to infer genome ploidy in *D. immitis*. The *x*-axis shows the normalised minor k-mer coverage, calculated as B/(A + B), where A and B are the coverages of the two alleles in a k-mer pair. The *y*-axis represents the total k-mer coverage (A + B), which corresponds to ploidy multiples (e.g., 2n, 3n, 4n, etc.). The colour gradient (log scale) indicates the frequency of k-mer pairs, with warmer colours (yellow) representing higher frequencies. The two main k-mer configurations identified are: AB (heterozygous diploid pairs, ~94%) and AAB (triploid-like k-mers, ~6%). The dominant AB signature, highlighted in yellow at the lower-right corner, strongly supports a diploid genome organisation, with a minor proportion of AAB suggesting potential local duplications or sequencing artefacts. The estimated haploid coverage (1n) is ~58×, as indicated on the side histogram.

**Figure 3:**
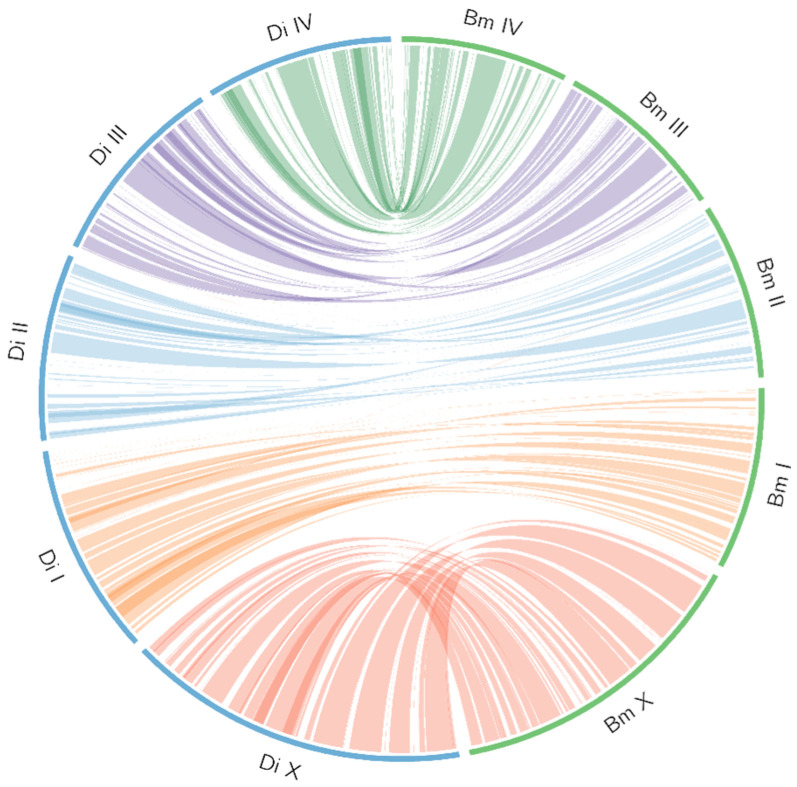
Circos plot illustrating genome-wide syntenic relationships based on single-copy orthologs between *Dirofilaria immitis* (light blue ideograms) and *Brugia malayi* (green ideograms). The coloured ideograms represent chromosomes of *D. immitis* (Di): Sex chromosome X (red): PGA_scaffold1__18_contigs__length_27,526,700. Autosomal chromosome I (orange): PGA_scaffold2__19_contigs__length_17,113,741. Autosomal chromosome II (blue): PGA_scaffold4__18_contigs__length_15,025,590. Autosomal chromosome III (purple): PGA_scaffold3__11_contigs__length_15,667,105. Autosomal chromosome IV (green): PGA_scaffold5__7_contigs__length_15,145,155. Black ideograms denote the corresponding chromosomes of *B. malayi*. Coloured ribbons within the circle illustrate orthologous connections, reflecting conserved synteny across homologous chromosomes.

**Figure 4:**
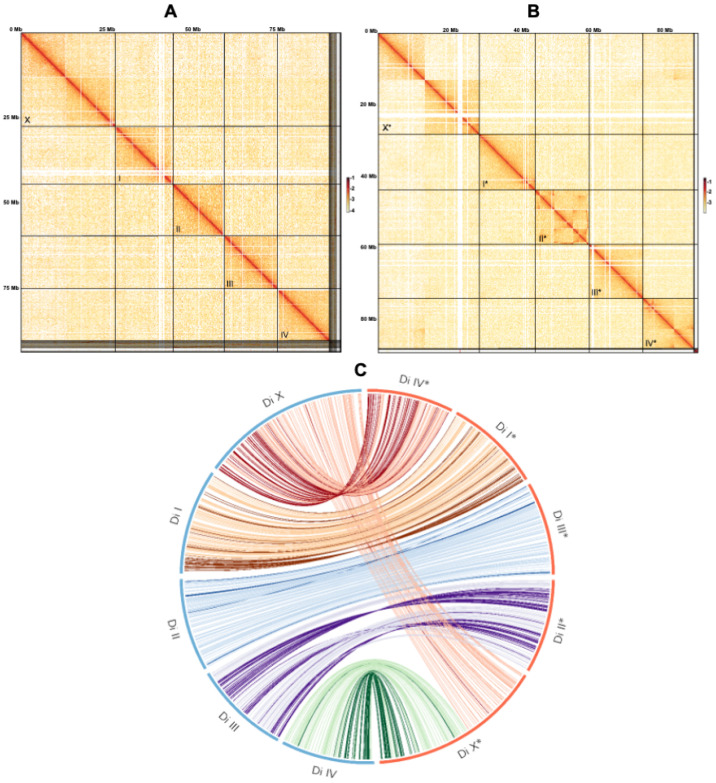
Chromosome conformation capture (Hi-C) contact maps for two *Dirofilaria immitis* genome assemblies. (**A**) The Hi-C contact map for the present *D. immitis* genome (Table 1)—assembled de novo—displays chromatin interaction frequencies across the genome of *D. immitis*. Each square along the diagonal corresponds to an individual chromosome-scale scaffold, revealing clear intra-chromosomal contact domains and well-resolved chromosomal architecture. The contact intensities (from white to deep red) represent the frequency of interactions between genomic regions, with higher frequencies shown in darker red. Strong diagonal signals indicate high internal connectivity, consistent with chromosome-scale contiguity. Contacts were rendered at 50 Kb resolution, and the balanced normalisation method was used. The x- and y-axes display the cumulative genetic distance in Mb along the genome. The scaffolds (chromosomes) representing *D. immitis* (Di) are: PGA_scaffold1 (X chromosome; 27.5 Mb; top left); PGA_scaffold2 (chromosome I; 17.1 Mb); PGA_scaffold3 (chromosome III; 15.7 Mb); PGA_scaffold5 (chromosome IV; 15.1 Mb); and PGA_scaffold4 (chromosome II; 15.0 Mb). (**B**) The Hi-C contact map for a previously published *D. immitis* genome assembly, guided by the reference genomes of *Brugia malayi* and *Onchocerca volvulus*—related filarioid species indicated distinct structural and configurational variation with respect to that displayed in panel (**A**). (**C**) Circos plot illustrating variation in the assembly between the present genome (represented in panel (**A**)) and a previously published genome of *D. immitis* (represented in panel (**B**)). For details, see Section 4.2).

The genome of *D. immitis* was ~94 Mb in size (*n* = 84 scaffolds)—consistent with the estimate from a k-mer analysis (Figure 1) and ~6 Mb larger than the genome of *B. malayi* (Table 1 and Table S1)—and was inferred to be diploid with limited heterozygosity (0.6%) (Figure 2). The chromosomal scaffolds representing *D. immitis* (Di) matched chromosomes I, II, III, IV and X of *B. malayi* (Figure 3). Interestingly, a comparative analysis of this *D. immitis* genome assembly revealed a distinction in the X-chromosome in the Hi-C map from that of a recently published genome (dimmitis_WSI_2.2; see Section 4.2) (Figure 4). To assess the validity of this difference, we calculated *p*-values for Hi-C support across X-chromosome to estimate the continuity of the assemblies. The difference between our assembly (*p*-value: 0.0055; genomic regions 12–13 Mb and >13–14 Mb) and dimmitis_WSI_2.2 (*p*-value: 0.94; same regions) supported the validity/quality of our assembly (Table S2).

The difference in percentage of repetitive sequences (9.7% for *D. immitis* and 18.3% for *B. malayi*) is likely due to the application of latest RepeatModeler v2.0.4 to the *D. immitis* genome sequence (Table 1). Despite this difference, the number of protein-coding genes (*n* = 11,852; Table 1) predicted using RNA-seq data (cf. Table S3) was similar to that of *B. malayi* (*n* = 11,350; Table 1). The statistics relating to genes, including proportion of genes in the genome, gene size, length of coding sequences (CDS), number of exons, lengths of exons and introns, were similar between *D. immitis* and *B. malayi* (Table 1). A BUSCO completeness of 93.6% is typical for a near-complete gene set of a nematode (Table 1). The vast majority of genes were annotated (Table S4) using multiple databases (see Section 4); only 789 genes were not annotatable.

### 2.2. Identification of Predictors of Essential Genes in D. immitis

We extracted 26 features for protein-coding genes in the nuclear genome of *D. immitis* (Table S5). These features had previously been identified as strong predictors of gene essentiality within *C. elegans* and *Dr. melanogaster* [7,8] and between these two species [13]; we also assessed the “evolutionary conservation” of the 26 features across 15 other eukaryotic species.

**Figure 5:**
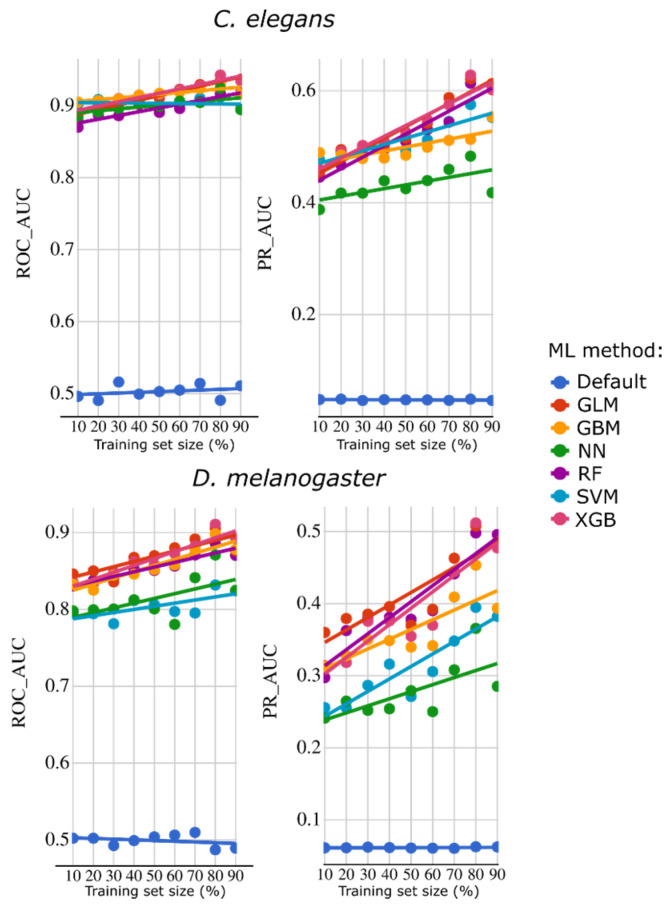
Machine learning (ML) performance metrics—receiver operating characteristic area under the curve (ROC-AUC) and precision-recall area under the curve (PR-AUC)—for the prediction of essential genes in *Caenorhabditis elegans* (**top row**) and *Drosophila melanogaster* (**bottom row**), using gene features available from *Dirofilaria immitis*. Six ML models were assessed: Gradient Boosting Machines (GBM), Generalised Linear Models (GLM), Neural Networks (NN), Random Forest (RF), Support-Vector Machines (SVM), and Extreme Gradient Boosting (XGB).

Before predicting essential genes in *D. immitis*, we evaluated the predictive performance of these 26 features for *C. elegans* and *Dr. melanogaster* using ML approaches with a sub-sampling framework for training, testing and evaluation (ROC-AUC and PR-AUC metrics). For *C. elegans* (Figure 5), ROC-AUC values exceeded 0.87 for all six ML models tested (GBM, GLM, NN, RF, SVM and XGB), reaching ~0.93 for GBM and XGB. PR-AUC values were usually >0.4, approaching 0.6 for the top-performing models (GBM and XGB) when trained with 90% of the data. For *Dr. melanogaster* (Figure 5), ROC-AUC values were >0.8, reaching ~0.9 for the best-performing models (GBM, GLM, RF and XGB). PR-AUC performance varied depending on the algorithm, ranging from ~0.3 to ~0.5 for the leading models (GBM, RF and XGB) when trained on 90% of the data.

Among the 26 features, the most informative predictors of gene essentiality in both *C. elegans* and *Dr. melanogaster* were OrthoFinder_species and exon count, followed by two subcellular localisation predictors (nucleus and cytoplasm) and GC content. Using the best-performing models (GBM and XGB, trained on *C. elegans* and *Dr. melanogaster* features), predictions for *D. immitis* indicated that 406 of 11,852 genes had a probability ≥ 0.5 of being essential, while 6180 had a probability < 0.1. More than 99.9% of these 406 ‘high-priority’ predictions had orthologues in *C. elegans*, and 57.7% were single-copy genes based on OrthoFinder v.2.2.6 results. These candidate genes, ranked by their probability of being essential, are provided in Table S6.

### 2.3. Association Between Essential Genes and Transcription Profiles

**Figure 6:**
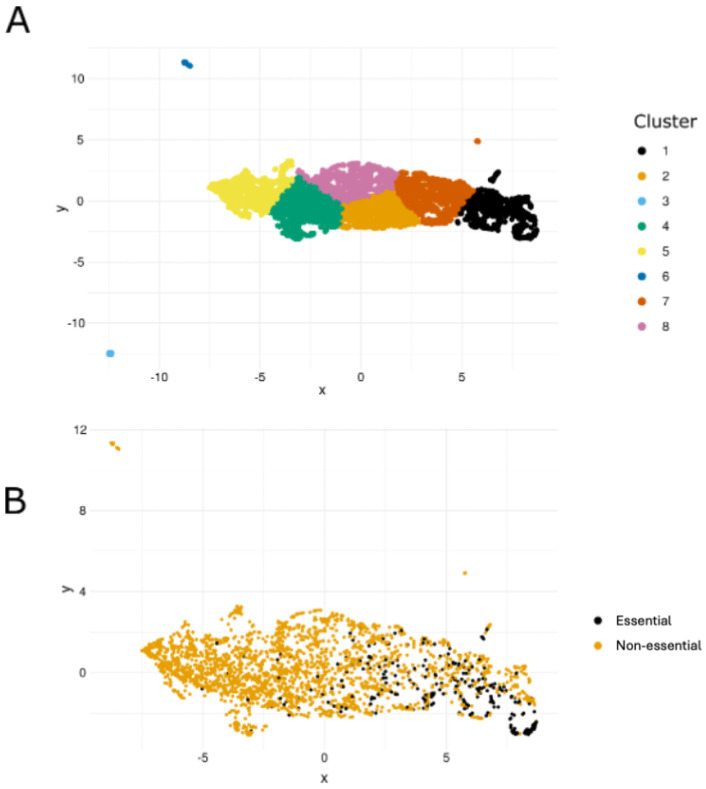
Transcriptional landscape of *Dirofilaria immitis* genes and their association with predicted essentiality. (**A**) UMAP clustering of 4700 genes consistently transcribed across all 25 RNA-seq samples available, showing distinct clusters with shared transcriptional profiles. (**B**) The same UMAP projection with an overlay of 282 predicted essential genes (black) and 2152 predicted non-essential genes (dark yellow), revealing their distribution across transcriptional clusters. This figure indicates associations between gene essentiality and specific transcriptional signatures in *D. immitis*.

To investigate the relationship between gene essentiality and transcription in *D. immitis*, we generated UMAP clusters from expression profiles across 25 distinct samples (Figure 6). After filtering out genes not expressed in all samples, 4700 genes (39.4%) remained. Of these, 282 of the 406 ‘high-priority’ predicted essential genes (69.5%) were retained, with most clustering together (Figure 6). In contrast, only 2152 of the 6180 most likely non-essential genes (~34.8%) remained, and these largely clustered apart from the essential genes (Figure 6).

Analysis of transcription in seven L3-stage samples showed that 363 of the 406 predicted essential genes (89.4%) had transcripts per million (TPM) values of >10, compared with 2424 of 6180 (~39.2%) of the non-essential set (cf. Table S7). To validate this finding, we independently confirmed the clustering of essential genes in *C. elegans* using its annotated essentiality data [7]. Clustering based on pooled RNA-seq data from 25 samples produced a similar pattern: following filtering, 282 of 406 (69.4%) predicted essential genes were retained and predominantly clustered together, whereas only 2152 of 6180 (34.8%) of the non-essential genes remained and grouped separately from the essential.

### 2.4. Essential Genes of D. immitis Are Inferred to Be Involved Predominantly in Ribosome Biogenesis, Translation, RNA Binding/Processing and Signalling

Gene ontology (GO) enrichment analysis for the prioritised list of the top-406 essential genes of *D. immitis* inferred molecular functions (*p* < 10^−10^) including ribosome/chromatin/cytoskeleton structure, protein dimerisation as well as ribonucleotide/cyclic compound binding; biological processes (*p* < 10^−8^) included ribosome biogenesis, ncRNA/RNA and/or nitrogen-compound metabolic processing; and cellular components (*p* < 10^−37^) nucleosome/chromatin/chromosome, ribosomes/ribonucleoproteins (intracellular non-membrane-bound organelles). Pathway enrichment indicated that orthologues of many of these genes were significantly associated (*p* < 10^−14^) with ribosome assembly (GTP hydrolysis, joining of the 60S ribosomal subunit and formation of free 40S subunits), translation initiation (eukaryotic and cap-dependent) and signalling/regulatory pathways (L13a-mediated translation silencing of ceruloplasmin expression, SRP-dependent co-translational protein targeting to membrane and nonsense-mediated decay).

### 2.5. Linking Essential Genes to Genome Locations, and Their Transcription to Cell Type or Tissue

**Figure 7:**
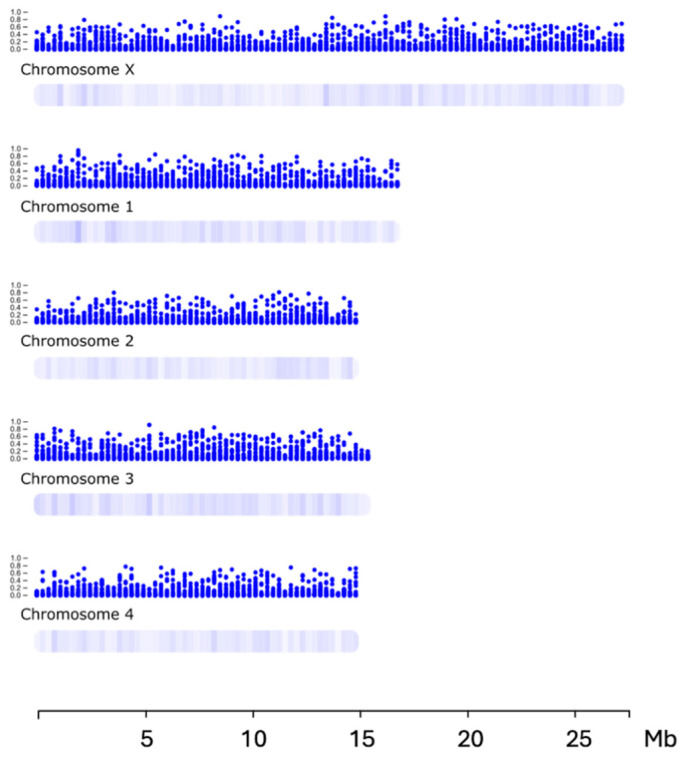
Predicted essentiality of individual *Dirofilaria immitis* genes, as determined by machine learning (ML), mapped to their respective genomic coordinates across major scaffolds representing the five chromosomes. Colour intensity reflects the probability of each gene being essential, enabling the visual identification of potential hotspots of essential genes within the genome.

First, we plotted the ML-based gene essentiality probabilities along individual *D. immitis* chromosomes (Figure 7). Most of the 406 ‘high-priority’ predicted essential genes (*n* = 286; 69%; probability > 0.5) were located on the four autosomes (PGA_scaffold2–5), without evidence of distinct positional clustering within each chromosome. Overall, essential genes occurred in “hotspots” that were relatively evenly distributed across all chromosomes. Chromosome X (PGA_scaffold1) harboured the largest proportion (*n* = 101; ~25%), followed by chromosome III (PGA_scaffold3; *n* = 95; ~23%) and chromosome I (PGA_scaffold2; *n* = 82; ~20%); only ~4% of essential genes were found in unplaced genomic fragments/debris.

**Figure 8:**
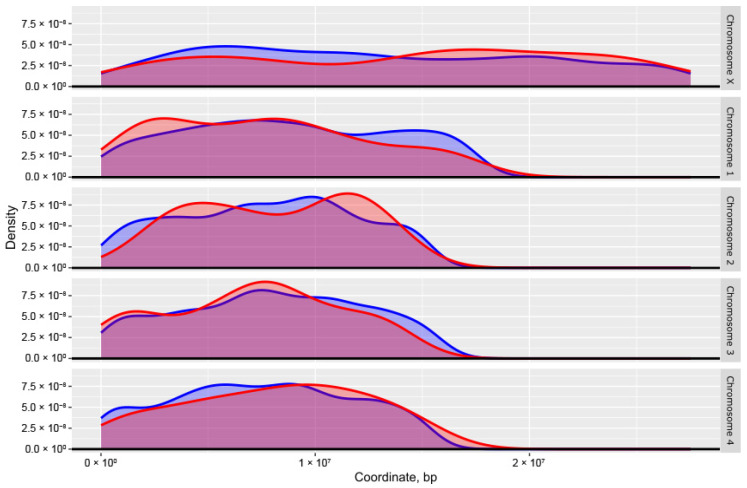
Chromosomal density distributions of the top-ranked predicted essential genes (*n* = 406; red) and non-essential genes (*n* = 6180; blue) in *Dirofilaria immitis*, based on machine learning (ML) classification. Density curves are plotted for each of the five major chromosomes, revealing similarities and differences in the spatial distribution of essential and non-essential genes across the genome.

Second, we compared distribution densities of the top 406 essential and 6180 non-essential genes across chromosomes (Figure 8). Slight density differences between essential and non-essential sets were detected on PGA_scaffold1, PGA_scaffold2 and PGA_scaffold4.

Third, using information available for *C. elegans* (see Section 4.3.4), we predicted the tissues and cell types in which essential genes are highly transcribed. Transcriptional mapping of *C. elegans* orthologues of the 406 top-priority *D. immitis* essential genes revealed strong representation in the germline (51 genes), somatic gonad precursors (35), body wall muscle (25) and sex myoblasts (20). Within the nervous system, 43 essential genes were highly expressed in amphid neurons with finger-like ciliated endings (AFD), 39 in asymmetric sensory neurons ASE/L and 24 in ASE/R. At the tissue level, 77 essential genes were abundantly transcribed in gonad, 61 in the hypodermis and 53 in body wall muscle.

## 3. Discussion

Extending previous work on gene essentiality in model ecdysozoans such as *C. elegans* and *Dr. melanogaster* [14], this study delivers the first large-scale ML-based prediction and characterisation of essential genes in the parasitic nematode *D. immitis*, accompanied by complementary analyses. We demonstrate a clear relationship between essentiality and transcriptional activity and establish a feature set for *D. immitis* that could aid future investigations of essential genes in related filarioid species.

To predict essentiality in *D. immitis*, we applied 26 features previously shown to be informative for *C. elegans* and *Dr. melanogaster* [13] and introduced two additional features linked to sequence conservation and transcription level, which also supported accurate predictions in these model organisms. This strategy identified genomic, conservation and expression attributes central to predicting essentiality, in line with earlier studies (e.g., (e.g., [13,14]). Such features can be readily derived from genomic and transcriptomic resources, and we also identified high-ranking candidate essential genes based on attributes that appeared specific to *D. immitis*. Nonetheless, these predictions will require experimental confirmation through gene knockout or knockdown approaches. Some predictive markers recognised in *C. elegans* and *Dr. melanogaster*, such as histone modifications (e.g., H3K4me3, H3K27me3; [7,8]), could not be assessed here due to the lack of equivalent data for *D. immitis*.

We propose a strong association between essentiality and transcriptional profile. Predicted essential genes in *D. immitis* clustered according to expression patterns, with essential and non-essential genes generally separating (Figure 7). Consistently, data from *C. elegans* showed that most (≥80%) essential genes were expressed across all samples and stages, while non-essential genes tended to be expressed at low levels and excluded during filtering.

Comparative analysis of scRNA-seq data for *C. elegans* indicated that ~25% of essential gene orthologues in *D. immitis* were highly expressed in reproductive tract tissues (germline and associated cells). This suggests that essential proteins expressed in reproductive tissues merit further study of their functions, structures and interactions as entry points for anthelmintic target validation.

As outlined in the *Introduction*, biological and technical constraints fundamentally limit the ability to perform functional-genomic experiments in *D. immitis* and most other parasitic nematodes of animals on a meaningful scale. Consequently, there are insufficient experimental data to provide true essentiality labels for *D. immitis*, and generating such data is restricted by several factors. First, the life cycle of *Dirofilaria* is exceptionally long and requires two obligate hosts, making experimental manipulation logistically complex and virtually impossible on a large scale. Second, critical developmental stages cannot be maintained in vitro under physiological conditions that accurately replicate those within the host, precluding high-throughput genetic perturbation or viability assays. Third, gene-knockdown tools have not been fully developed and are not feasible for medium- to large-scale experiments—particularly for filarial worms—as the delivery of molecular reagents into infective or reproductive stages remains technically prohibitive. In addition, ethical and regulatory constraints increasingly limit the use of experimental infections in vertebrate hosts for validation. Collectively, these challenges mean that functional-genomic validation on the scale achievable in model organisms such as *C. elegans* is currently not feasible in *D. immitis*. Consequently, empirical calibration of thresholds separating essential from non-essential genes is not possible, and an absolute probability threshold cannot yet be defined with confidence. Therefore, genes were ranked according to their relative probability of essentiality across the 11,852 genes analysed, and a working threshold of 0.5 was applied solely to highlight the highest-priority, high-ranking candidates for future experimental investigation once enabling technologies become available.

Genes predicted to be essential were not more frequently located on autosomes than on the sex chromosome in *D. immitis*, in contrast to *C. elegans* [7]. Their relatively uniform distribution across chromosomes also differed from the patterns reported in *C. elegans* (enrichment near chromosome centres; [7]) and *Dr. melanogaster* (bias away from centromeres; [8]). These differences may reflect variations in genome organisation, centromere architecture or regulatory mechanisms (genetic *versus* epigenetic), as well as distinct aspects of species biology. GO and pathway analyses indicated that many *D. immitis* essential genes are involved in transcriptional regulation, RNA binding, ribosome biogenesis and/or translation initiation, consistent with findings in *C. elegans* and *Dr. melanogaster* [14].

Comparison of our *D. immitis* genome assembly with a recently published chromosome-scale assembly (Figure 4) suggested structural and configurational differences. In our assembly, we found no evidence for the alternative configuration reported previously, suggesting that these discrepancies may have arisen from reference-guided scaffolding or represent genuine population-level genomic variation. These findings highlight the importance of independent de novo assemblies and the integration of long-read sequencing with Hi-C data to resolve chromosomal structures and minimise artefacts that could compromise interpretations of genome organisation and gene content.

This genome-wide ML-based prediction of essential genes in *D. immitis* provides a framework for experimental validation to predict genes required for parasite survival. Given the persistent challenges of treatment and control, prioritising essential genes for laboratory study through computational pipelines is critical. AI-driven approaches, combined with expanding genomic and phenomic data sets, offer a means to accelerate both fundamental and applied research on essential genes and drug target discovery. We anticipate that the strategy applied here could be extended to other eukaryotic pathogens of medical and veterinary significance.

## 4. Materials and Methods

### 4.1. Nucleic Acid Sequence Data Sets

Genomic data for *D. immitis* were produced for Boehringer Ingelheim (Athens, GA, USA) by Phase Genomics (Seattle, WA, USA) or were accessed from public databases (Table S1). Chromatin conformation capture data was generated using the Phase Genomics (Seattle, WA, USA) Proximo Hi-C 4.0 Kit—which is a commercially available version of the Hi-C protocol [18]. Following the manufacturer’s instructions for the kit, intact cells representing adult individuals of *D. immitis* were crosslinked using a formaldehyde solution, digested using the restriction enzymes *Dpn*II, *Dde*I, *Hinf*l and *Mse*l, and proximity-ligated with biotinylated nucleotides to create chimeric molecules composed of fragments from different regions of the genome that were physically proximal in vivo, but not necessarily genomically proximal. Continuing with the manufacturer’s protocol, molecules were isolated with streptavidin beads and processed into an Illumina-compatible sequencing library. Sequencing was performed on an Illumina NovaSeq (Illumina Inc., San Diego, CA, USA), generating >302,000,000 read pairs (PE150 read pairs) per sample.

Most transcriptomic data were available publicly from previous studies or provided by Boehringer Ingelheim (Athens, GA, USA) (sample nos. 19–65; Table S3; cf. [19,20,21]). Some transcriptomic data were produced from fresh/frozen (–80 °C) *D. immitis* samples (nos. 1–18) sent frozen (–196 °C) from Boehringer Ingelheim (USA) to The University of Melbourne (Table S3). In brief, total RNA was isolated from individual worms using the TriPure Isolation Reagent (Sigma Aldrich, Burlington, MA, USA) and DNase-treated (TURBO DNA-free^TM^ kit, Thermo Fisher Scientific, Waltham, MA, USA). The size, integrity and amount of RNA were determined using a 4200 TapeStation System RNA ScreenTape Assay (Agilent Technologies, Waldbronn, Germany) and a Qubit^®^ 3.0 fluorometer RNA High Sensitivity Assay (Life Technologies, Carlsbad, CA, USA). Messenger (m)RNAs were individually purified from total RNAs using the Dynabeads^®^ mRNA Purification Kit (Thermo Fisher Scientific), and separate libraries constructed for paired-end short-read RNA-sequencing performed at the Australian Genome Research Facility (AGRF) in Melbourne, Australia, or BGI Hong Kong Company Limited.

### 4.2. Genome Assembly, Prediction of Repeats and Genes, and Comparative Analysis

The nuclear genome of *Dirofilaria immitis* (derived from a single male) was assembled and annotated using established computational workflows. For contig assembly and phasing, FALCON-Phase [22] was employed to correct probable phase-switching errors between primary contigs and alternate haplotigs, generating pseudohaplotype contig sets for each phase. Hi-C reads were aligned to the phased assembly following the Phase Genomics Proximo Hi-C Kit protocol (https://phasegenomics.github.io/2019/09/19/hic-alignment-and-qc.html; accessed on 10 August 2025). Reads were mapped using BWA-MEM [23] with −5SP and −t 8 options; PCR duplicates were flagged using Samblaster v.0.1.26 [24] and excluded. Alignments were filtered using Samtools v.1.21 [25] with the −F 2304 flag to remove non-primary and secondary alignments. Chromosome-scale scaffolds were generated using the Proximo Hi-C genome scaffolding platform (Phase Genomics), following an established single-phase procedure [26]. As in the LACHESIS method [27], a Hi-C contact frequency matrix was computed from the aligned read pairs, normalised by the number of restriction sites (*Dpn*II, *Dde*I, *Hinf*I and *Mse*I) on each contig. Scaffolds were constructed to optimise expected contact frequencies and overall Hi-C patterns. Approximately 20,000 Proximo runs were performed to optimise scaffold number and structure. Manual curation was conducted using Juicebox [28,29] to correct misassemblies. A second FALCON-Phase run was then used to correct residual phase-switching errors at the scaffold level. Metadata from this step enabled construction of a fully phased, diploid, chromosome-scale genome using a purpose-built script. To characterise genome architecture, genome size and heterozygosity were estimated using GenomeScope v2.0 and Smudgeplot v0.2.2 [30]. Mitochondrial and *Wolbachia* sequences were identified and removed using BLAST+ v2.12.0 [31].

To assess genomic assembly contiguity, we analysed Hi-C read pairs that capture physical contacts between chromosomal regions. Only pairs with reliable mapping quality and correct orientation were retained, and these were ordered consistently along each chromosome. The genome was partitioned into overlapping 1 Mb bins, offset by 0.5 Mb, to ensure full coverage, and the number of Hi-C contacts linking each bin to its adjacent bin was counted. As a baseline, a background distribution was defined from all inter-chromosomal contacts, representing the expected frequency of non-adjacent interactions, and each observed contact count was compared against this background using a z-score and two-sided *p*-value, assuming normally distributed background counts.

For genome annotation, first, custom repeat models, inferred from the genome using RepeatModeler v.2.0.4 (RepeatModeler Open-1.0. 2008–2015; https://www.repeatmasker.org; accessed 20 January 2025), were masked in the assembled genome employing RepeatMasker v.4.1.5 [32]. Then, protein-coding gene models were predicted from the masked genome using BRAKER3 [33] incorporating all available RNA-seq data sets as evidence (Table S3). RNA sequence reads were trimmed to achieve a mean PHRED score of ≥30 and a length of ≥60 bp; adapter sequences were removed using Trimmomatic v0.36 [34].

Subsequently, the quality of protein-coding genes was assessed using the program table2asn [35] from the National Center for Biotechnology Information (NCBI) (Bethesda, MD, USA) and BUSCO v5.1.2 [36]. Genes that met the quality standard set by NCBI were annotated using databases including InterPro v5.51–85.0 [37], UniProt/SwissProt (cf. [38]), KEGG (for eukaryotes) [39] and NCBI non-redundant (nr) [40]; the files for submission to NCBI were prepared using both custom scripts and table2asn.

Single-copy orthologues (SCOs) and one-to-one orthologs were identified using OrthoMCL v2.5.4 [41]. Genome synteny was analysed using Circos v0.68.8 [42]. First, gene content and order were compared between *D. immitis* (this study) and *B. malayi* (GCA_000002995.5), and then with an independent genome assembly for *D. immitis* [43]. For the synteny analysis, the latter genome was annotated ab initio using BRAKER3.

### 4.3. Predicting and Ranking Essential Genes by ML and Associated Analyses

We employed a structured workflow to predict essential genes and to explore their transcriptional and functional characteristics (cf. Figure S1). The workflow comprised four main phases: (i) feature extraction and selection; (ii) model training, prediction and ranking; (iii) analysis of transcriptional clustering; and (iv) integration of genomic, tissue-level and functional annotations.

#### 4.3.1. Feature Extraction and Selection

From the 11,852 genes predicted from the genome of *D. immitis* (Boehringer Ingelheim), we extracted 9569 features linked to DNA sequences, protein sequences and subcellular localisation (inferred using DeepLoc 1.0 [44]) following established methods [7,8]. In total, 26 features were selected for *D. immitis* and used for ML-based prediction and evaluation of gene essentiality; these features are listed and described in Table S5. These features, which were also predictive in *C. elegans* and *Dr. melanogaster* [13], include the “OrthoFinder_species” feature describing protein-sequence conservation among species. Predicted proteomes representing 15 eukaryotic species (Table S5) from divergent branches of the Tree of Life [45] were obtained from Ensembl, and orthologous groups were identified using OrthoFinder v.2.2.6 [46] with default parameters. The number of species represented within each orthologous protein group was then determined for *C. elegans*, *Dr. melanogaster* and *D. immitis*.

Features were selected by random subsampling from 10% to 90% of data representing “essential” or “non-essential” genes (in 10% increments) based on consensus between ElasticNet (α = 0.5) and ensemble Sparse Partial Least Squares (SPLS) using glmnet and *enspls* in R [26]. The selected features were used to train six ML models: Gradient Boosting Machine (GBM), Generalised Linear Model (GLM), Neural Network (NN), Random Forest (RF), Support Vector Machine (SVM) and Extreme Gradient Boosting (XGB; *xgbTree*), implemented in the caret R package (version 7.0-1). Parameter tuning and five-fold cross-validation were performed, and models achieving the highest ROC-AUC were retained. After subsampling, the remaining data (90% to 10%) were used to evaluate final model performance using ROC-AUC and PR-AUC. Each algorithm was then retrained on 100% of each dataset to generate the final predictions.

To prevent data leakage, orthologous genes were represented once per species, and all training and test partitions were orthology-aware and non-overlapping. The same six algorithms were subsequently applied in the *D. immitis* prediction pipeline using five-fold stratified cross-validation and grid-search hyperparameter tuning (details provided in the public code repository: https://zenodo.org/records/15368523). For reference datasets, cross-validated ROC-AUC values ranged from 0.90 to 0.94 and PR-AUC from 0.55 to 0.68, consistent with benchmarks from earlier model-organism analyses [7,8,13].

#### 4.3.2. Predicting and Ranking of Essential Genes

We assessed the individual and collective powers of the 26 selected features to predict essential genes in *C. elegans* and *Dr. melanogaster* using six distinct ML models (GBM, GLM, NN, RF, SVM and XGB) [7,8]. The best-performing models, GBM and XGB, based on ROC-AUC and PR-AUC metrics, were then applied to predict essential genes in *D. immitis*. Corresponding orthologs in *C. elegans* and *Dr. melanogaster* were identified for all *D. immitis* genes using OrthoFinder v.2.2.6 [46]. All *D. immitis* genes were ranked in descending order according to their predicted probabilities of being essential, as defined by the two best-performing ML models.

A working probability threshold of 0.5 was applied to define ‘high-priority’ predictions, representing a balanced operating point between precision and recall in the cross-validated models. This threshold was selected for practical ranking and prioritisation purposes rather than to impose a strict binary classification of essential versus non-essential genes. Performance across alternative thresholds (0.3, 0.5 and 0.7) was evaluated to illustrate the trade-offs between precision and recall. Feature-importance analyses were conducted to identify the most influential predictors contributing to model output, and the resulting ranked list of genes (Table S6; cf. Figure S2) was used to guide subsequent analyses.

#### 4.3.3. Analysis of Transcription and Clustering

Transcription levels of individual genes in TPM were inferred using the program RSEM v.1.3.3 [47] and bowtie2 v.2.3.2 [48]. Genes were assigned to eight clusters, according to their transcription profiles in different developmental stages (samples) of *D. immitis.* For this analysis, we used unsupervised clustering, employing uniform manifold approximation and projection (UMAP; “umap” package for R), with random initialisation. Following assignment, gene clusters were displayed using the “ggplot2” package in R.

#### 4.3.4. Genomic, Tissue and Functional Annotation of Essential Genes

For each gene of *D. immitis* inferred from our prediction/annotation approach, we associated the genomic location in the General Feature Format (GFF) annotation file with the corresponding ML-based probability of being essential. The probability of each gene being essential (defined by ML) was mapped to the five largest scaffolds (representing the chromosomes) of *D. immitis* using “chromoMap” for R.

We also inferred cell types and tissues in which the “essential gene orthologs” of *D. immitis* predicted were abundantly transcribed in *C. elegans*. For these analyses, we used existing scRNA-seq data available (Cao_et_al_2017_vignette.RData file; cf. [49]). Genes were then subjected to GO and pathway enrichment analyses based on their respective orthologs in *C. elegans* using g:Profiler ((https://biit.cs.ut.ee/gprofiler/page; accessed on 2 February 2025) [50] and the Reactome Pathway Database(https://reactome.org/; accessed on 20 January 2025) [51]. For GO enrichment, reported *p*-values were corrected using their default correction method g:SCS at 0.05 significance (https://biit.cs.ut.ee/gprofiler/page/docs; accessed 20 March 2025). For pathway enrichment, reported *p*-values were FDR-corrected at 0.05 significance (https://reactome.org/userguide/analysis; accessed 2 February 2025).

## Figures and Tables

**Table 1:** Statistics for the genomes of *Dirofilaria immitis* and *Brugia malayi*. Metrics include genome size, scaffold statistics (N50 and N90 with corresponding L50 and L90 values), G+C content, proportion of repetitive and genic sequences (exonic and intronic), and protein-coding gene characteristics (number, size, exon/intron structure and coding GC content). BUSCO (Benchmarking Universal Single-Copy Orthologs) scores indicate the completeness of the genome. Percentages represent proportions of conserved nematode genes that were complete, or complete plus fragmented.

Features	*D. immitis* (This Study)	*B. malayi* (GCA_000002995.5)
Genome size (bp)	94,056,743	88,235,797
Number of scaffolds	84	197
N50 (bp); L50	15,667,105; 3	14,214,749; 3
N90 (bp); L90	15,025,590; 5	13,467,244; 5
Genome GC content (%)	27.8	28.5
Repetitive sequences (%)	9.7	18.3
Exonic proportion; incl. introns (%)	16.5; 56.0	18.6; 55.5
Number of protein-coding genes predicted (isoforms)	11,852 (14,247)	11,350 (not reported)
Mean; median gene size (bp)	4447; 3101	4315; 3015
Mean; median protein-coding gene length (bp)	1292; 933	1317; 1095
Mean exon number per protein-coding gene	9.4	9.1
Mean; median exon length (bp)	140; 129	161; 140
Mean; median intron length (bp)	375; 243	354; 231
Coding G+C content (%)	38.4	39.1
BUSCO completeness: complete; complete + fragmented (%)	93.6; 94.4	98.9; 99.1

## Data Availability

The complete genome assembly is available in the NCBI (National Center for Biotechnology Information) database under accession numbers PRJNA1246367 and SAMN47776112. All data and code are available via https://zenodo.org/records/15368523.

## Supplementary Materials

The supporting information can be downloaded at https://www.mdpi.com/article/10.3390/ijms26209923/s1.
