# Supplementary material for: Genome-Wide Inference of Essential Genes in Dirofilaria immitis Using Machine Learning
Source: Int J Mol Sci. 2025 Oct 12;26(20):9923. doi: 10.3390/ijms26209923 (PMC12562366; doi:10.3390/ijms26209923)
Supplement: Supplementary file 1 [file ijms-26-09923-s001.zip › Figure S1.pdf]

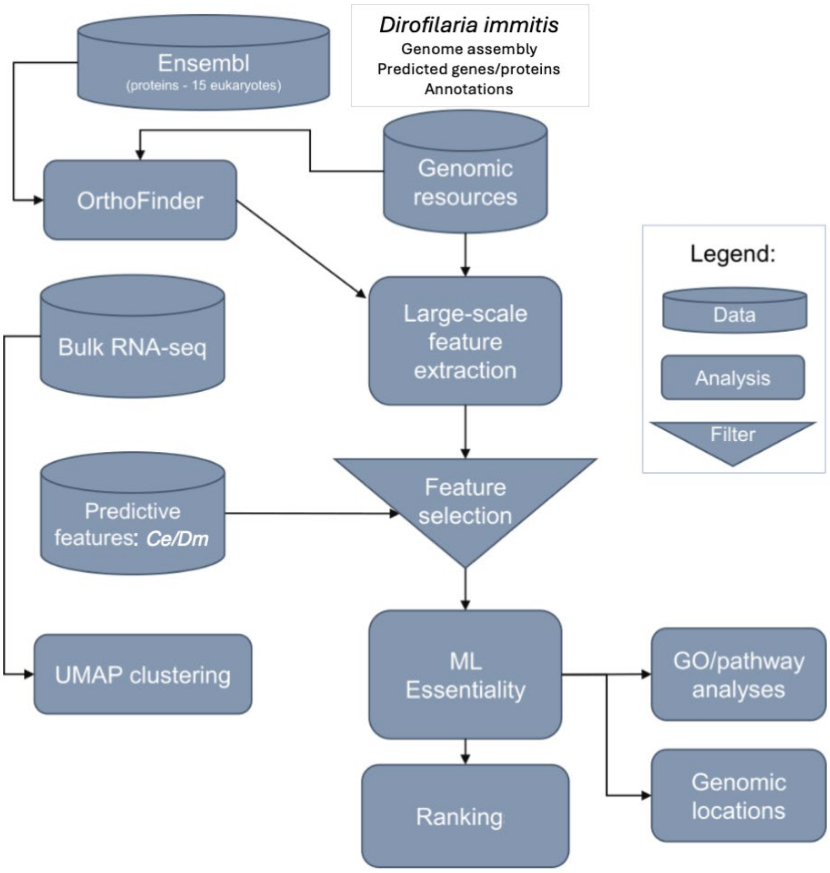

Figure S1. The workflow used for the prediction and proritisation (ranking) of essential genes in *Dirofilaria immitis* using machine learning (ML) and complementary analyses (see section 2. *Materials and methods*). A range of features was extracted from *D. immitis* genes, and key predictive features from *Caenorhabditis elegans* and *Drospohila melanogaster (Ce/Dm)* were used to train ML models and predict essential genes. The relationship between essentiality and transcription was investigated by clustering analyses; other complementary analyses included gene ontology (GO)/pathway enrichments and the mapping of gene essentiality probability to genomic locations.
