# Supplementary material for: Genome-Wide Inference of Essential Genes in Dirofilaria immitis Using Machine Learning
Source: Int J Mol Sci. 2025 Oct 12;26(20):9923. doi: 10.3390/ijms26209923 (PMC12562366; doi:10.3390/ijms26209923)
Supplement: Supplementary file 1 [file ijms-26-09923-s001.zip › Figure S2.pdf]

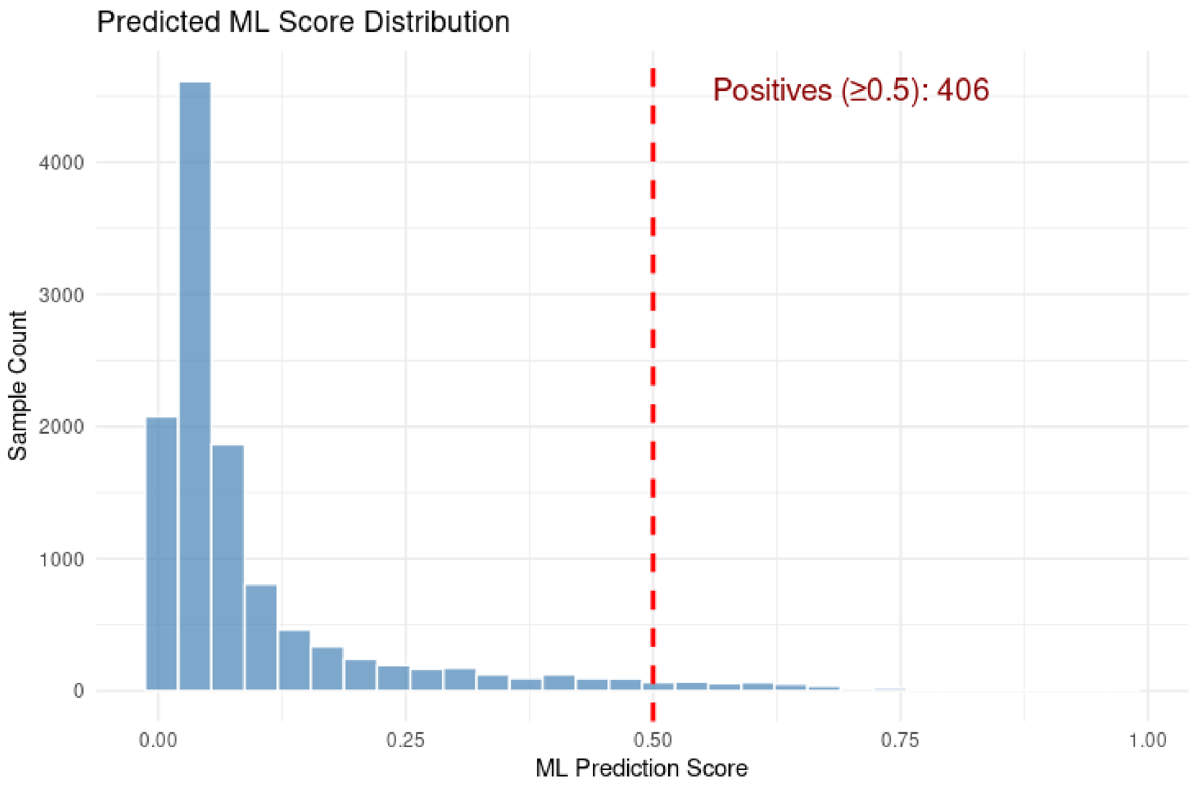

Figure S2. The diagram illustrates the selected prediction score of 0.5 (red dotted line) used as a threshold to prioritise essential genes for *D. immitis* in the present study.
