# Supplementary material for: Genome-Wide Inference of Essential Genes in Dirofilaria immitis Using Machine Learning
Source: Int J Mol Sci. 2025 Oct 12;26(20):9923. doi: 10.3390/ijms26209923 (PMC12562366; doi:10.3390/ijms26209923)
Supplement: Supplementary file 1 [file ijms-26-09923-s001.zip › Table S5_rbg24 copy.pdf]

Table S5. Features (n = 26) that were used to predict essential genes in *Dirofilaria immitis*; these features are predictive of essential genes within and between *Caenorhabditis elegans* and *Drosophila melanogaster*, and accompanying list (below) of 15 eukaryotic species from which proteomes were inferred and ortholog groups identified with respect to the inferred proteome for *D. immitis* – using the program OrthoFinder (see Subsection '*4.3.1. Feature extraction and selection*' for details).

Features	Descriptions	Sources
OrthoFinder_species	Orthologs in other species	OrthoFinder analysis
exons	Number of exons	Extracted from gene models
exons_total_length	Total length of exons	Extracted from gene models
Cytoplasm	Subcellular localisation	DeepLoc analysis
Mitochondrion	Subcellular localisation	DeepLoc analysis
Nucleus	Subcellular localisation	DeepLoc analysis
AAC_S	Protein sequence feature	Extracted using protR*
APAAC_Pc2.Hydrophobicity.2	Protein sequence feature	Extracted using protR*
CTDC_secondarystruct.Group1	Protein sequence feature	Extracted using protR*
CTDD_prop4.G2.residue0	Protein sequence feature	Extracted using protR*
CTDD_prop4.G2.residue25	Protein sequence feature	Extracted using protR*
CTriad_VS153	Protein sequence feature	Extracted using protR*
CTriad_VS431	Protein sequence feature	Extracted using protR*
CTriad_VS613	Protein sequence feature	Extracted using protR*
DC_HA	Protein sequence feature	Extracted using protR*
DC_MP	Protein sequence feature	Extracted using protR*
DC_MS	Protein sequence feature	Extracted using protR*
DC_VF	Protein sequence feature	Extracted using protR*
Geary_CHOC760101.lag7	Protein sequence feature	Extracted using protR*
Moran_CHAM820102.lag7	Protein sequence feature	Extracted using protR*
GC	DNA sequence feature	Extracted using codonW
kmer_3_GCT	DNA sequence feature	Extracted using rDNAse*
PseKNC_3_Xc1.CCC	DNA sequence feature	Extracted using rDNAse*
PseKNC_5_Xc1.CGT	DNA sequence feature	Extracted using rDNAse*
PseKNC_5_Xc1.GCT	DNA sequence feature	Extracted using rDNAse*
TACC_Nucleosome.lag2	DNA Sequence feature	Extracted using rDNAse*

*For further information about those sequence features, refer to the R packages protR (https://cran.r-project.org/web/packages/protr/vignettes/protr.html) and rDNAse (https://cran.r-project.org/web/packages/rDNAse/vignettes/rDNAse.pdf) documentations.

Eukaryotic species	Link; version or code
Arabidopsis thaliana	https://www.arabidopsis.org/; TAIR10
Aspergillus fumigatus	https://ngdc.cncb.ac.cn/databasecommons/database/id/1357; CADRE
Caenorhabditis elegans	https://www.ncbi.nlm.nih.gov/datasets/genome/GCF_000002985.6/; WBcel235
Danio rerio	https://www.ncbi.nlm.nih.gov/datasets/genome/GCF_000002035.6/; GRCz11
Drosophila melanogaster	https://www.ensembl.org/Drosophila_melanogaster/Info/Inde; BDGP6
Fusarium graminearum	https://fungi.ensembl.org/Fusarium_graminearum/Info/Index; RR1
Homo sapiens	https://www.ncbi.nlm.nih.gov/datasets/genome/GCF_000001405.26/; GRCh38
Mus musculus	https://www.ncbi.nlm.nih.gov/datasets/genome/GCF_000001635.20/; GRCm38
Ovis aries	https://www.uniprot.org/proteomes/UP000002356; UP000002356 9940
Plasmodium berghei	https://www.ncbi.nlm.nih.gov/datasets/genome/GCF_900002375.1/; PBANKA01
Plasmodium falciparum	https://protists.ensembl.org/Plasmodium_falciparum/Info/Index; 3D7 (ASM276v2)
Saccharomyces cerevisiae	https://www.ensembl.org/Saccharomyces_cerevisiae/Info/Index; R64-1-1
Schizosaccharomyces pombe	https://www.ncbi.nlm.nih.gov/datasets/genome/GCF_000002945.1/; ASM294v2
Toxoplasma gondii	https://www.ncbi.nlm.nih.gov/datasets/genome/GCA_000149715.2/; TGGT1
Trypanosoma brucei	https://protists.ensembl.org/Trypanosoma_brucei/Info/Index; TryBru_Apr2005
